# Longitudinal patterns of cytokine expression at the individual level in humans after laparoscopic sleeve gastrectomy

**DOI:** 10.1111/jcmm.15309

**Published:** 2020-04-26

**Authors:** Uriel Trahtemberg, Fares Darawshe, Ram Elazary, Isaac Ginsburg, Michael Beil, Peter Vernon van Heerden, Sigal Sviri

**Affiliations:** ^1^ General Intensive Care Unit Hadassah – Hebrew University Medical Center Jerusalem Israel; ^2^ Medical Intensive Care Unit Hadassah – Hebrew University Medical Center Jerusalem Israel; ^3^ Surgery Department Hadassah – Hebrew University Medical Center Jerusalem Israel; ^4^ Institute for Dental Sciences Hebrew University Faculty of Dental Medicine Jerusalem Israel; ^5^ Philosophisch‐Theologische Hochschule der Pallottiner Institute of Health Sciences Vallendar Germany; ^6^ Present address: Critical Care Department St. Michael’s Hospital Toronto ON Canada

**Keywords:** clustering analysis, cytokines, human, inflammation, laparoscopic sleeve gastrectomy, longitudinal, trauma

## Abstract

The study of the human response to injury has been hampered by the inherent heterogeneity in the models and methods used. By studying a standard injury longitudinally, using individual patient‐level analysis, we endeavoured to better describe its dynamics. We analysed clinical variables, clinical laboratory and plasma cytokines from 20 patients at five time points. Clustering analysis showed two prototype patterns of cytokine behaviour: a concordant type, where cytokines behave the same way for all patients (notably IL‐0 and TNFα), and a variable type, where different patterns of expression are seen for different patients (notably IL‐8, IL‐6 and IL‐1RA). Analysis of the cytokines at the individual patient‐level showed a strong four‐way correlation between IL‐1RA, GCSF, MIP‐1β and MCP‐1. As it holds for most patients and not just on average, this suggests that they form a network which may play a central role in the response to gastro‐intestinal injuries in humans. In conclusion, the longitudinal analysis of cytokines in a standard model allowed the identification of their underlying patterns of expression. We propose that the two prototype patterns shown may reflect the mechanism that separates the common and individual aspects of the injury response.

## 1 INTRODUCTION

Injuries to the human body produce acute local and systemic inflammatory responses, aimed at containing and healing the damage.^1^ The systemic consequences of these inflammatory responses vary depending on the severity of the injury.^2^ Cytokines serve as immune mediators in these injuries, signalling and regulating the immune response. They are also used as biomarkers for prediction of outcome and are sought as targets for therapeutic interventions.^1^, ^3^, ^4^ Unfortunately, these efforts have been hampered by our incomplete understanding of the connection between local injury and the systemic inflammatory response.^2^ While several previous studies have analysed cytokines and their correlation with the systemic inflammatory response, sepsis and trauma, several shortcomings have raised questions regarding the reliability of some of these studies, such as the study of heterogeneous populations and injuries that then underwent varying iatrogenic interventions. Some studies used a few cytokines or only single mediator classes; cytokines act in a network; thus, analyzing only a small number of cytokines is of limited value. Another important reason for the lack of consensus in the human cytokine literature relates to the significant variability in the sampling times relative to the injury, given the fact that the acute cytokine responses are rapid and by 48 hours most of the changes are over.^5^, ^6^ Also, the changes from baseline within cytokine networks appear to be very important,^1^, ^7^ making the sampling strategy crucial in order to observe these changes. All these problems raise concerns that many of these studies would be limited in their ability to reflect the pathological process at hand (for a representative sample see Ref. 8, 9, 10, 11, 12). While these past efforts have been pioneering, the analysis of such complex scenarios with the methods used has led to limited success when trying to collectively generalize and synthesize them. In order to try and deal with the heterogeneity of the immune response in humans, previous efforts have tried to tackle this by looking for response subpopulations with the use of large scale investigations followed by multivariate analysis in stable conditions,^13^ with the use of in vitro approaches,^14^ or using pattern recognition for defined disease ‘snapshots’.^15^ Still, acute, dynamic diseases in vivo in humans are much more difficult to study. Important advances have been made trying to account for the individual variability inherent in such cases by relying on data from clinical trials (for example see Ref. 16). While randomization allows these trials to assume heterogeneity is equally partitioned, the heterogeneity itself is very high (patients, treatments, disease progression, sampling time, etc).

On another front, several animal studies have demonstrated that analysis of time‐dependent cytokines patterns can provide important insights into the mechanisms of inflammation.^17^ However, being single genotype and phenotype studies, these studies lack the variability encountered in humans, and their utility for clinical application is not clear.^18^, ^19^, ^20^

In order to overcome some of the problems described, we set out to study a standard injury in humans. Laparoscopic sleeve gastrectomy (LSG) represents such a standard tissue injury model of the inflammatory response to abdominal gastro‐intestinal injury. It reproduces the same inflammatory stimulus in a relatively homogenous group of patients who begin at a steady state. In essence, it allows to study a clinical event with conditions that approach a controlled laboratory experiment. Previous studies have examined the long term changes in inflammatory markers following bariatric surgery,^21^, ^22^, ^23^ yet none of these studies examined the changes in inflammatory markers and cytokines in the perioperative period, nor was the analysis done in a personalized manner. Also, the number of cytokines previously examined was limited, and they were not clinically correlated. In this study, we aimed to analyse the cytokine patterns and their development over time, at the individual level, as well as their correlation to the patients’ clinical characteristics and outcome.

## 2 MATERIALS AND METHODS

### 2.1 Experimental design

We performed a prospective, descriptive and analytic study in a group of 20 patients with morbid obesity who underwent laparoscopic sleeve gastrectomy (LSG) as a model for the inflammatory response to abdominal injury. It was performed at the Hadassah Medical Center in Jerusalem, Israel, over a nine‐month period. Informed consent was obtained from all participants prior to inclusion in the study. The study and protocol were approved by the Hadassah—Hebrew University Medical Center Institutional Ethics Committee (approval number 0624‐15‐HMO), in keeping with the principles of the Declaration of Helsinki and ICH‐GCP E6, and according to the regulations on human research by the Israeli Ministry of Health. Inclusion criteria included: age between 18 and 65 years and fulfilment of the surgical criteria for bariatric surgery; specifically, multiple unsuccessful attempts of weight loss and a body mass index (BMI) greater than 40 kg/m^2^, or greater than 35 kg/m^2^ if there are associated comorbidities. Exclusion criteria included: refusal or withdrawal of consent, failure to obtain blood samples and baseline haemoglobin level less than 9 gr/dL. Previous studies have shown that even among patients after major trauma or severe illness, most of the dynamics in cytokine levels occurred in the first 48 hours^5^, ^24^, ^25^, ^26^, ^27^; therefore, sampling must be performed early. Accordingly, the times were chosen to try and sample the expected changes in mediator blood levels, together with the logistic constraints of drawing blood from patients. A 6‐mL blood sample was obtained from each patient at each of the following five time points: (a) pre‐operatively, immediately after insertion of an intravenous line (defined as 0 hours); (b) immediately post‐operatively, upon arrival at the recovery room; (c) three hours after the 2nd sample; (d) the morning of post‐operative day one (POD 1) (24 hours); and (e) the morning of POD 2 (48 hours). Every blood sample included 2 tubes: an EDTA tube for Luminex assays and a heparin tube used for clinical biochemistry. Blood samples used for Luminex assay were immediately stored at 4°C. The samples were centrifuged within 20 minutes (5 minutes centrifugation time at 300 g relative centrifugal force at a temperature of 4°C.), and the plasma was frozen. Samples 1 to 3 were centrifuged and frozen at −20°C in the recovery room with on‐site equipment, and then transferred on the same day to the laboratory for permanent storage at −80°C. Samples 4 and 5, which were taken in the ward, were directly centrifuged and stored at −80°C in the laboratory. In order to minimize degradation and standardize results, all samples underwent up to 2 freeze and thaw cycles, with each specific cytokine measured after the same number of cycles for all samples. Cytokine data are missing for one patient due to technical problems. Clinical data were recorded every time a blood sample was obtained, including systolic blood pressure (SBP), diastolic blood pressure (DBP), heart rate (HR), oxygen saturation (SaO_2_) and fraction of inspired oxygen (FiO_2_), body temperature, respiratory rate (RR) and visual analog scale (VAS, 0‐10) pain scale. Patients were observed for surgical complications throughout the admission, such as surgical site and IV cannula site infections.

### 2.2 Surgical technique

All surgeries were performed by the same surgeon (RE), using an identical technique. The average surgical time (defined from start to end of anaesthesia care) was 71 minutes, with a SD of 21 minutes (range 47 to 135 minutes). This SD was driven by two cases where there were unexpected delays after starting anaesthesia care before the actual surgery began, when excluded, the SD drops to 14 minutes and the average to 66 minutes. The surgical approach was laparoscopic with the use of four trocars. Gastrectomy was performed after releasing the major gastric curvature, from a distance of 5 cm from the pylorus to the gastro‐oesophageal junction, adjusted to a 42‐Fr intra‐gastric bougie catheter. The staple line was tested for homeostasis and after that for leakage using intra‐gastric methylene blue, after pylorus obstruction. The specimen of the major gastric curvature was removed through the trocar incision. Haemostasis was secured, and all trocars removed under vision. An intraperitoneal suction drain was placed in all patients and was routinely removed 3 days after surgery.

### 2.3 Data collection and storage

A paper case report form was kept for each patient, containing clinical information including demographic data, identification information, age, gender, past medical history and current active medical problems, as well as medications and vital signs throughout the perioperative study period. This and all other study data were then recorded digitally using de‐identified serial numbers into an Excel table (Microsoft). Once recorded, only the study lead had access to the identification table.

### 2.4 Cytokine measurements

The Human High Sensitivity Cytokine Premixed kit (R&D systems, Inc) was used to determine the level of IL‐1β, IL‐2, IL‐6, IL‐8, IL‐10 and TNFα. To determine the level of GCSF, IL‐1RA, IL‐2R, LAP, MCP‐1, MIP‐1β, MMP‐2, MMP‐3, MMP‐9, sIL‐2R and TGFβ, the Procartaplex Human Multiplex Immunoassay (Affymetrix—eBioscience) was used. Histone H3 was measured using ELISA (Mybiosource.com). All assays were performed according to manufacturer's instructions. The assay plates were read using a Luminex analyzer (MagPix, Luminex Corporation).

### 2.5 Clinical biochemistry

Serum levels of sodium, potassium, creatinine, urea, lactate dehydrogenase (LDH), ferritin, lactate and creatine phosphokinase (CPK), as well as a complete blood count (CBC) were obtained using the hospital's clinical laboratory services.

### 2.6 Statistics

Power analysis: Biancotto et al^28^ investigated the inter‐individual variation of cytokine levels. Based on these data and the expectation of a fourfold increase of cytokine levels (based on our own preliminary data), the minimal sample size needed to detect a statistically significant difference (*P* < .001) with a power of 0.95 is 10 individuals. The low threshold for the alpha error is necessary due to correction for multiple tests, and the sample size was doubled to account for biological and methodological heterogeneity.

The raw data were prepared as follows: (a) values that were below or above the limit of detection of the assay were set to the corresponding limit of detection. (b) Outliers were defined as 3 interquartile ranges (IQR) above the 75th or below the 25th quartile (analysed per experimental batch). Outliers were winsorized, replacing them with the corresponding outlier limit. (c) Missing values were imputated by replacing the 1st sample with the 2nd, the 5th sample with the 4th, and the 2nd, 3rd or 4th samples with the average of the two neighbours. (d) If a sample had more than one missing value, it was excluded from analysis. Samples were also excluded if 3 or more values were zero or beyond the upper or lower limits of detection of the assay. *P*‐values below .05 were considered statistically significant throughout. Statistical analysis was carried out with JMP Pro v13.2 (SAS Institute). The mixed model analyses of the variables studied were set up using the patients as random effects and the sampling times as fixed effects. Statistical significance was tested using the Wald test for the random effects’ covariance estimates and the F test for the fixed effects. Comparison between sampling times in the model was performed using Tukey's HSD test. Clustering was done using the self‐organizing map which is an unsupervised clustering algorithm that bears resemblance to k‐means clustering and takes in account the shape of the data being clustered and not just the proximity between the points (ie tries to preserve the topological properties of the original data).^29^ This is useful in order to cluster together individuals with similar trajectories over time. Clustering algorithms require pre‐specification of the number of clusters to be fitted. In order to decide how many clusters are underlying the data, we chose the largest possible number of clusters that yielded more than one patient per cluster, as a cluster of one patient is trivial statistically. In most cases, this provided three clusters; therefore, for simplicity and clarity, throughout the article we used 3 clusters for all parameters. For clustering, the data were prepared using the Johnson transformation. For the correlation analysis at the individual level, we tested all cytokine pairs, separately for every patient, using Spearman's ρ. Then, the *P*‐values of all patients for a given pair were combined using Fisher's method. In order to control for multiple testing, we used Benjamini and Hochberg's false discovery rate correction with a Q level of 0.05. This was repeated for the clinical laboratory and vital signs together versus the cytokines.

## 3 RESULTS

**FIGURE 1:**
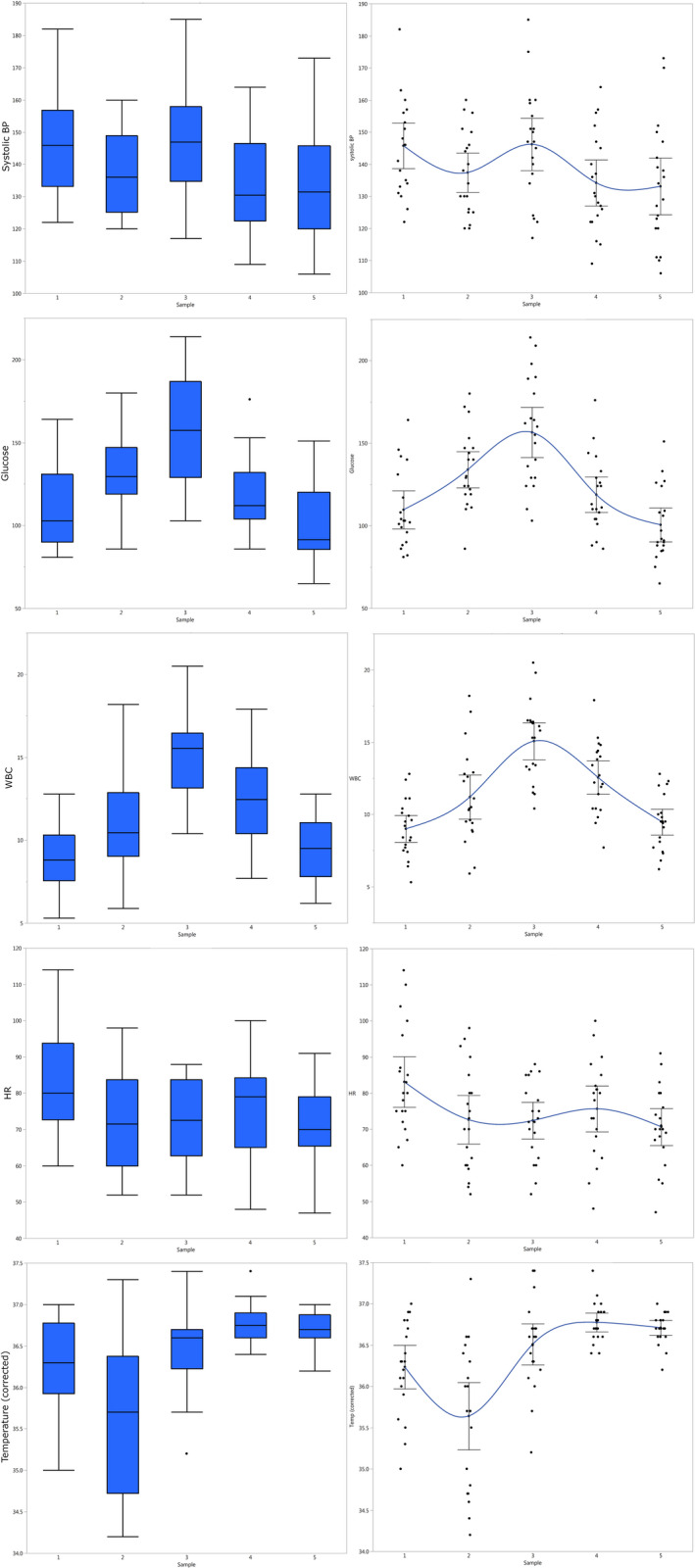
Vital signs and clinical laboratory trends. The distribution of the different variables is shown over the five sampling times for all patients (n = 20). In the left column, they are shown as box plots. The box is bound by the 3rd quartile at the top and the 1st at the bottom, encompassing the interquartile range; the line inside the box represents the median; the whiskers are drawn extending 1.5 times the interquartile range from the top and bottom of the box; outliers beyond this range are shown as dots. In the right column, all the measurements are shown as dots, with a fitted curve passing through the averages, connecting them; the whiskers encompass the 95% confidence interval of the mean. The units are as follows: blood pressure in mmHg, heart rate (HR) in beats per minute, temperature (Temp) in degrees Celsius, glucose in mg/dL and white blood cells count (WBC) in 10^9^/L

The demographic characteristics of the study population were as follows: average age 38.5 years (range 18‐60); average BMI 44 kg/m^2^ (range 35‐64); 11 of the 20 patients were male; 45% had hypertension, 35% diabetes mellitus, 25% hyperlipidaemia and 55% had nonalcoholic steatohepatitis. Two post‐operative complications were observed in the study, both minor: one case of phlebitis (treated by removal of the IV catheter) and one case of local surgical site infection (which was treated with local lavage). Systolic blood pressure, blood glucose, white cell counts, heart rate and temperature are shown in Figure 1. There is a clear difference in most measurements between the sample immediately after surgery (sample 2) and the one three hours post‐op (sample 3). Ostensibly, the former shows residual effects of anaesthesia while the latter shows the influence of rebound due to pain and injury. The next two samples (post‐operative days [POD] 1 and 2, which correspond to samples 4 and 5, respectively) show a gradual return to baseline.

**FIGURE 2:**
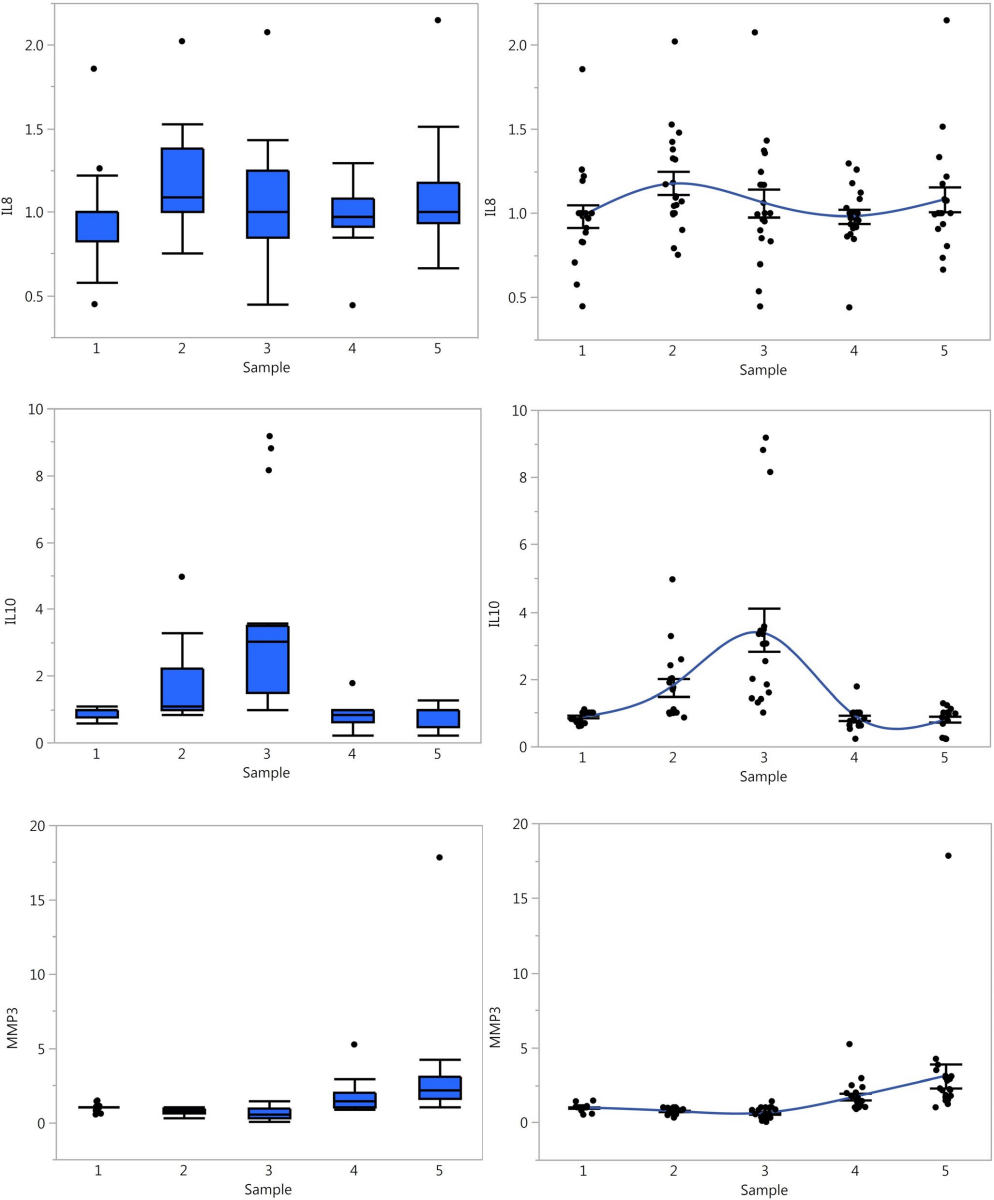
Distribution of IL‐8, IL‐10 and MMP‐3 over time. The distribution of the different cytokines is shown over the five sampling times for all patients (n = 19). In the left column, they are shown as box plots. The box is bound by the 3rd quartile at the top and the 1st at the bottom, encompassing the interquartile range; the line inside the box represents the median; the whiskers are drawn extending 1.5 times the interquartile range from the top and bottom of the box; outliers beyond this range are shown as dots. In the right column all of the measurements are shown as dots, with a fitted curve passing through the averages, connecting them; the whiskers encompass the 95% confidence interval of the mean. The values of the cytokines have been row normalized using the median of each patient's five samples, in order to compare between patients and across different experiment batches

For the cytokines, the responses to the surgery are best described by their dynamics. As shown in Figure 2, there are fast‐responding cytokines, such as IL‐8, which increase immediately post‐op; intermediate‐speed cytokines, such as IL‐10, which peak 3 hours post‐op; and late cytokines, such as MMP‐3, which increase later on PODs 1 and 2. The responses of the whole set of cytokines tested are shown in Figure S1.

We performed mixed model analyses of the variables to discern which component of the responses was due to variabilities in the patients (a random effect) and which component was due to a common response to the procedure, expressed by the progression over sampling times (a fixed effect). Table 1 shows the results of this analysis. In the ‘patient’ and ‘sample’ columns, ‘+’indicates that the effect significantly contributed to the model's ability to explain the results (defined as *P* < .05). The column ‘residual variance’ shows how much variance is left unaccounted by the best‐fit model of the regression analysis (ie how much of the results the model cannot explain). For most of the vital signs and laboratory measurements, the model could account for a considerable portion of the results’ variance, indicating that most of the results can be explained using the two specified effects. In contrast, for the cytokines, there are much fewer instances where the effects are significant; except for MMP‐9, the model did not manage to appropriately explain the results. The full results of the pairwise comparisons between sampling times for all variables in the model are shown in Figure S2.

**TABLE 1:** Regression analysis using a mixed model

Variables	Patient	Sample	Residual variance (% of total, rounded)
SBP	+	+	66%
DBP	+		71%
MAP	+	+	65%
HR		+	76%
Temperature		+	100%
Pain		+	83%
Glucose	+	+	65%
WBC	+	+	48%
Neutrophilia	+	+	53%
HGB	+	+	16%
PLT	+	+	10%
Creatinine	+		2%
CRP		+	76%
Ferritin	+		100%
GCSF	+		100%
Histone 3		+	96%
IL‐1RA		+	100%
IL‐6	+		100%
IL‐8	+		100%
IL‐10		+	89%
MCP‐1		+	100%
MIP‐1β		+	100%
MMP‐3		+	95%
MMP‐9	+	+	56%
TGFβ	+		100%
TNFα		+	100%

**FIGURE 3:**
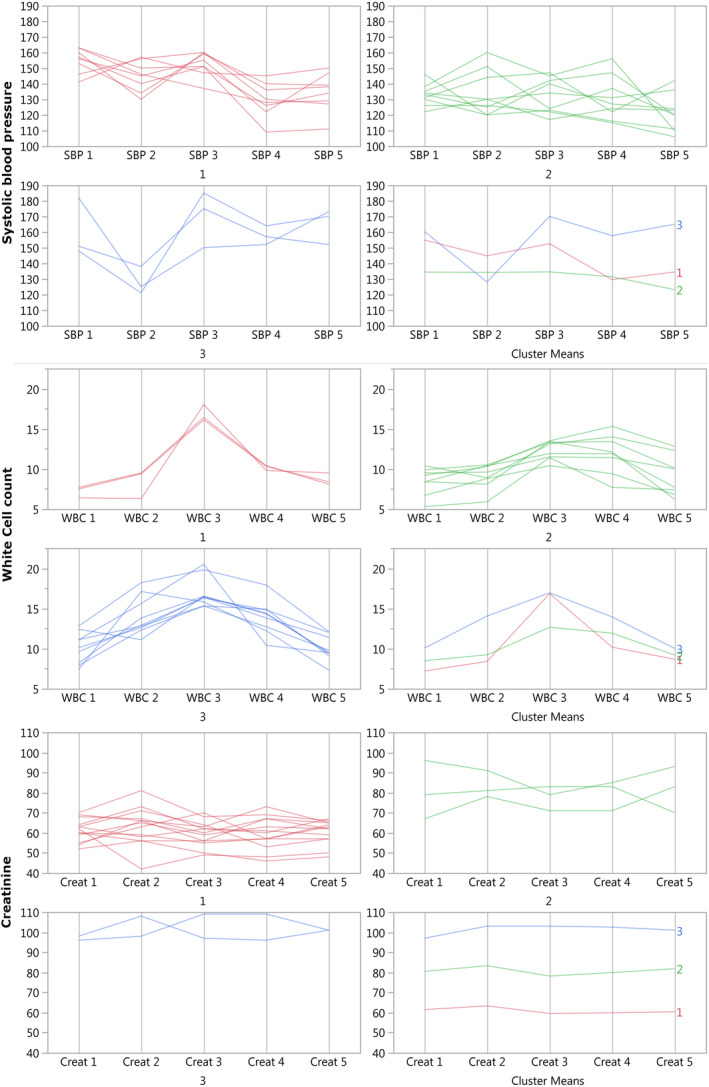
SBP, WBC and creatinine clusters. The parameters were plotted so that the ‘*Y*’ axes represent the absolute values measured and the ‘*X*’ axes represent the five sampling times. Every line represents a single patient, and all patients were included (n = 20). The red, green and blue plots represent the three different clusters found, numbered 1 to 3, for every parameter. Each parameter was clustered separately; therefore, the patient allocation in the clusters is not the same for the different parameters (ie the patients in cluster 1 for the SBP are not necessarily the same as in cluster 1 of the WBC). The cluster means represent the averaged response of each cluster. The SBP is measured in mmHg, the WBC in 10^9^/L and the creatinine in µmol/L

As the whole cohort analysis could not explain the cytokine results, we set out to analyse the results differently, at the individual level. As can be seen in Figure 3 (for the vital signs and laboratory measures), clustering analysis can enhance our insight into the physiological processes affecting the results. The individual variability of the responses is clearly shown in a way that is not expressed with cohort‐level statistics. For SBP for example, the patients in the green cluster (cluster number 2) start slightly hypertensive and continue that way. The patients in the red and blue clusters (numbers 1 and 3) both start hypertensive; the red ones stay hypertensive and then decrease by about 20 mm Hg at POD 1, while the blue ones exit the surgery almost normotensive and then jump back to hypertension. Presumably, the ‘green’ patients are slightly hypertensive at baseline and stay that way; the ‘red’ patients have controlled hypertension but did not take their medications on the day of the surgery and then restarted them post‐operatively; and the ‘blue’ patients have uncontrolled hypertension which is reduced under anaesthesia and then returns to its baseline high level. These presumptions were confirmed when the patients’ charts were retrospectively reviewed in light of these results, providing strong evidence for the use of this clustering technique to discover underlying physiological trends. As a counter example, the WBC clusters all show a pattern of increase and then decrease, albeit with different kinetics. Finally, the creatinine clusters show that there were no changes from baseline. The clustered trajectories of the rest of the vital signs and clinical laboratory results are shown in Figure S3.

**FIGURE 4:**
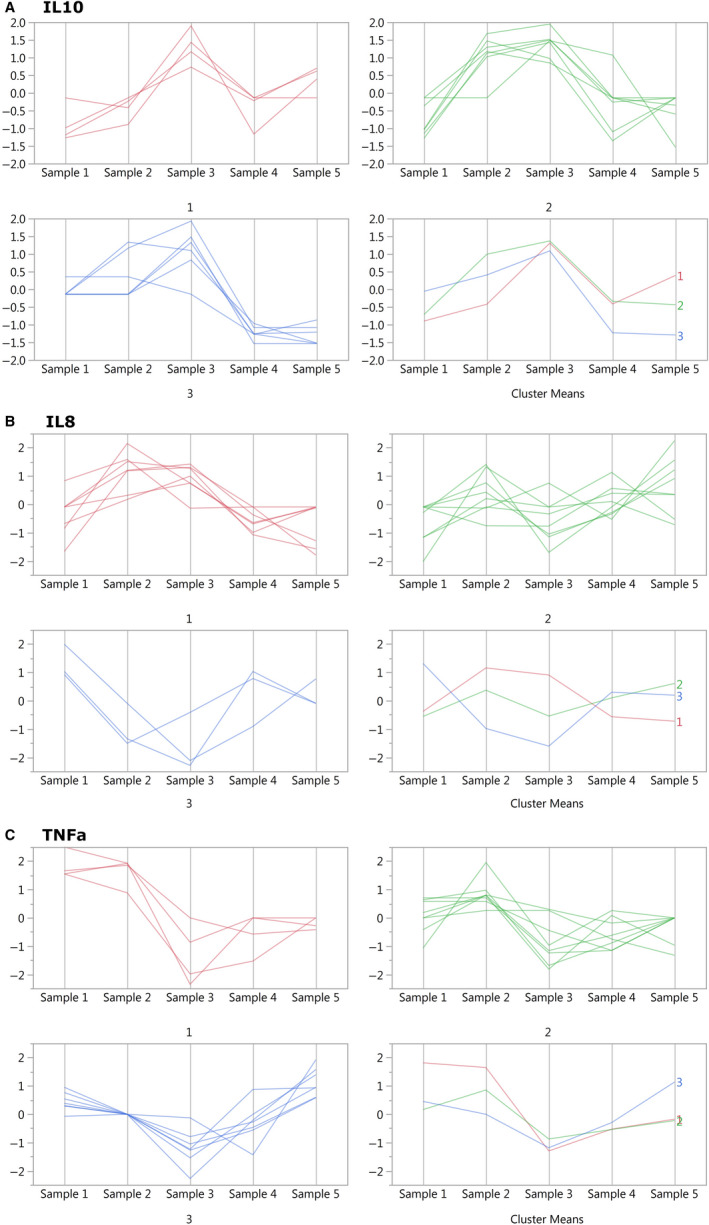
IL‐10, TNFα and IL‐8 clusters. The parameters were plotted so that the ‘*Y*’ axes represent the median‐normalized cytokine values, per patient (as in Figure 1), and the ‘*X*’ axes represent the five sampling times. Every line represents a single patient, and all patients were included (n = 19). The red, green and blue plots represent the three different clusters found, numbered 1 to 3. Each cytokine was clustered separately; therefore, the patient allocation in the clusters is not the same for the two cytokines (ie the patients in cluster 1 for IL‐10 are not necessarily the same as in cluster 1 of IL‐8). The cluster means represent the averaged response of each cluster. Normalization per patient allows comparing the fold change in expression over time between the different patients but does not show absolute levels

When analyzing the cytokine data at the level of individual trajectories using the clustering algorithm, two general types of responses were observed: concordant ones, where all patients followed the same pattern of response, and personal ones, where different patients could follow different patterns of response. Figure 4A shows the response of IL‐10, where all patients responded the same way, with a steady increase that peaks at sample time 3 and then decreases gradually towards baseline over the next two days. Even though the kinetics are slightly different, the patterns of the response are clearly the same for all patients. Figure 4B shows IL‐8, where, in contrast, there are three distinct clusters of response patters: cluster 1 (red) shows an increase followed by a decrease; cluster 3 (blue) a decrease followed by an increase; and cluster 2 (green) shows variations about the baseline. TNFα (Figure 4C) behaves similarly to IL‐10 but with changes in the opposite direction; this inverse relation between the behaviour of TNFα and IL‐10 exemplifies a pro‐ and anti‐inflammatory paired equilibrium. The clustered responses for all the rest of the cytokines are shown in Figure S4. The summary of the cytokines’ behaviour is as follows: CRP, histone H3, MIP‐1β, MMP‐3, MMP‐9, IL‐10 and TNFα show a concordant response; GCSF, IL‐1RA, IL‐6, IL‐8, MCP‐1 and MMP‐2 show two to three different clusters of response; and sIL‐2R and TGFβ are unclear due to high levels of experimental noise.

Given the technical limitations of the platforms used for the quantification of biomarkers in research settings, the comparison of absolute cytokine levels of cytokines across different experimental runs is fraught with biases and potential errors.^7^, ^30^, ^31^ Therefore, we use the normalized change over time, the temporal trends, instead of absolute numbers; this harnesses the platform's linear response which is verified in every experiment with the use of calibration curves. This method is also supported by the variability in absolute levels among patients, which makes directly comparing absolute levels less informative. Given the use of normalized temporal patterns, in order to detect whether patients have the same behaviour across cytokines (ie for a given patient all cytokines increase, or all cytokine decrease), we classified the patterns observed as increasing, decreasing or ‘no major longitudinal changes’ (Figure 4 and Figure S4). Using this classification, we found no consistent trends among any given patient, including when analyzing separately pro‐inflammatory and anti‐inflammatory cytokines. The small sample size precludes the analysis of subgroups of response types.

We also set out to determine the associations between the results. Table 2 shows the results for the cytokines vs vital signs and clinical laboratory. This is a description of the relationship between the biochemical underpinnings of the inflammatory response and the resulting clinical phenotype. All vital signs and clinical laboratory measures were analysed for correlation with all of the cytokines, at the individual level. Then, the results of all of the patients were combined, followed by adjustment for multiple tests using the false discovery rate procedure, as described in the materials and methods. Table 2 presents the results that were statistically significant after the adjustment, indicating significant correlation between the pair of variables. Out of the 8 statistically significant pairs, six are inflammatory or acute phase reactants (CRP, ferritin, TNFα, histone and MCP‐1) and the other two are MMPs, all of which are directly related to the amount of tissue damage. Four of the vital signs/clinical laboratory results were an expression of inflammation (leukocytosis and neutrophilia), and three were signs affected by inflammatory mediators (glucose, RR and SBP). Table 3 shows the statistically significant associations between the cytokines themselves, using the same technique as for Table 2. This is a description of the immune reaction elicited by the surgery. Of special note is the quadrilateral correlation between IL‐1RA, GCSF, MIP‐1β and MCP‐1; each one is related to the other pairwise, creating a network.

**TABLE 2:** Statistically significant correlations between cytokines and clinical variables at the individual level

Vital sign/ clinical laboratory	Cytokine	Adjusted *P*‐value
Glucose	MMP‐3	.0006
WBC	TNFα	.0009
Respiratory rate	CRP	.0164
SBP	Histone H3	.0338
WBC	MMP‐9	.0351
%NE	MCP‐1	.0306
WBC	Ferritin	.0377
HGB	CRP	.0499

**TABLE 3:** Statistically significant correlations between cytokines at the individual level

Cytokine pair	Adjusted *P*‐value
IL‐1RA ‐ GCSF	<.0001
MIP‐1β ‐ GCSF	<.0001
MIP‐1β ‐ IL‐1RA	<.0001
MIP‐1β ‐ MCP‐1	<.0001
TGFβ ‐ MIP‐1β	.0002
MCP‐1 ‐ IL‐1RA	.0002
sIL‐2R ‐ GCSF	.0003
MMP‐9 ‐ MMP‐3	.0004
sIL‐2R ‐ MCP‐1	.0005
TNFα ‐ IL‐1RA	.0008
TGFβ ‐ GCSF	.0033
TNFα ‐ MCP‐1	.0034
MCP‐1 ‐ GCSF	.0043
TNFα ‐ MIP‐1β	.0084
MMP‐3 ‐ CRP	.0103
sIL‐2R ‐ MIP‐1β	.0147
TGFβ ‐ IL‐1RA	.0145
IL‐10 ‐ Histone H3	.0235
TNFα ‐ sIL‐2R	.0334
IL‐10 ‐ GCSF	.0383
TNFα ‐ TGFβ	.0388
MMP‐2 ‐ CRP	.0412
MMP‐9 ‐ MMP‐2	.0406

## 4 DISCUSSION

One of the challenges of personalized medicine lies in identifying molecular targets that show variations between different patients, which are amenable for intervention, and that are likely to affect the clinical course of the patient. Almost by definition, this requires analysis of the data at the individual level. In this investigation, we tried to advance that goal by studying the cytokine, vital signs and laboratory responses to a standard injury (LSG) and analyzing the results from a personalized perspective.

When studying the vital signs and clinical laboratory results, the regression model was able to explain a substantial part of the variability, yet provided little insight into the processes at hand. The cluster analysis, in contrast, uncovered a new layer of information that would have been lost otherwise, reflecting the physiological differences between different groups of patients. SBP showed clear subgroups of responses, while WBC showed different kinetics for the same response type. Regarding the cytokine analysis, the regression analysis failed to account for most of the variability. Cluster analysis, on the other hand, working at the individual level, opens a window into the dynamics of the cytokines. We would like to discuss the cytokine clustering results in three respects:

### 4.1 IL‐10 and TNFα

Their similar kinetics, but with opposite changes, play out in a way that clearly outlines our understanding of the expected immune response to injury. While these results might seem unsurprising given the current body of knowledge,^32^, ^33^ it should be noted that this knowledge comes largely from animal and basic science models.^34^, ^35^ In this study, we have been able to show IL‐10 and TNFα’s kinetics in detail, in response to an actual injury in humans. This demonstrates the actual occurrence in vivo of the behaviour that is expected, based on pre‐clinical or partial models. Although others have shown similar kinetics in other human models, these have been averaged results; our analysis was performed at the individual level. This means that the changes we show in IL‐10 and TNFα are not averaged courses, but rather that all patients respond in the same way; this carries more weight when trying to establish a universal description of the response to injury. In that sense, these results support the hypothesis that the anti‐inflammatory arm is an integral part of the immune response to injury, beginning together with the inflammatory arm and not in reaction to it.^36^

### 4.2 Individual‐level analysis

The past three decades have seen many studies on cytokines. Many times the results have been incongruent, difficult to understand or difficult to translate into clinical applications.^37^ A large number of the clinical initiatives based on cytokine data have had underwhelming results.^38^ This has caused many scientists and clinicians to steer away from the field and cast doubt on whether cytokine research is worthwhile for clinical applications. We propose that the problem is not that cytokines are not clinically useful, but rather we need to improve our methodologies. Previous studies had significant clinical and technical heterogeneity, which made it very difficult to compare and synthetize them, as described in the introduction. Indeed, in most cases, the data were analysed in a way that did not account for the actual complexity of the models studied. As a counterpoint, cytokine‐based clinical applications have been successful in cases where the clinical entity treated and the variables used to measure outcome were well defined, such as with anti‐TNFα for Rheumatoid Arthritis.^39^ It is in this context that we propose that not only should we apply more rigorous models and methods for cytokine studies, we should also apply more advanced analysis tools, beyond ‘standard statistics’. For example, in our study, if we were to compare average levels of IL‐8 at baseline vs 3 hours after surgery for all patients together (Figure 2), the conclusion would be that there is considerable variability, and, in average, there is no change between those two points. This is very different from the conclusion drawn from the results of our individual‐level analysis, which shows that there are 3 different subgroups of responses (Figure 4).

### 4.3 Concordant‐type vs personal‐type patterns

When studying immune responses to in vivo*,* one of the major challenges is that, while the macroscopic responses mounted are largely similar, there is significant inter‐personal variability at the molecular and cellular level. How can these be reconciled? Recent results, although in vitro, would seem to suggest that genetics can only explain a minority of the heterogeneity.^40^ One conclusion from our results is that this heterogeneity seems to have boundaries, as the cytokines are still constrained in their expression dynamics. They do not behave randomly, as their patterns fall within certain forms, as shown in Figure 4 and Figure S4. Still, this does not explain how patients have similar macroscopic results while having different molecular responses. One possible explanation is that the common macroscopic responses are be driven by the consensual (or concordant) cytokines, where all patients share the same cytokine pattern. In this scenario, these concordant‐type cytokines would be the ones driving the generic response to the injury, while the personal‐type cytokines would reflect the particularities of a specific injury or individual, modulating the final result. These two patterns may have been missed in the past due to dis‐synchronous sampling times, heterogeneous injuries and whole cohort‐level analysis. We have recently shown similar patterns of cytokine expression, demonstrating constrained heterogeneity, in another model of standard injury, bedside tracheostomy among critically ill patients. In that case, though, we did not observe consensual type patterns, most likely due to the decreased signal to noise (ie lower injury magnitude in the background of sicker patients) and increased heterogeneity of the patients.^41^ Recently, there have been significant advances towards recognizing the factors imparting some of the personal heterogeneity by the host (such as age and immunological history) and the environment (such as season of the year).^42^ Our results are another way to understand the mechanism of the coexistence of heterogeneity on top of a common injury response.

The correlation analysis presented was also performed in a way that preserves each patient's dynamics, as well as assessing these correlations over time, not just at single time points. We show that there is a core of four cytokines (IL‐1RA, GCSF, MIP‐1β and MCP‐1) that stand out in their reciprocal correlation. It has been shown that cytokines do not act on their own but rather in synergism, forming networks among themselves (as well as with cells and other mediators).^43^, ^44^ Other works that analysed cytokine networks found subsets of the same correlations between the same cytokines as we have found.^24^ All together, these results suggest that these four cytokines may play an important role in the response to gastro‐intestinal injury and bear specific investigation in future studies.

Our work has some limitations, including a small sample size that precluded the performance of more correlations; the use of clinical measurements and plasma biomarkers only; and the use of obese patients only. Nonetheless, it should be noted that the range of BMI in our cohort was very large, from 35 to 64 kg/m^2^. The fact that we still saw consensual cytokine responses from the slightly obese to the severely morbid obese lends reassurance that this response is not affected by obesity.

In conclusion, individual‐level, longitudinal analysis of LSG as a standard model of abdominal gastro‐intestinal injury shows that cytokine responses can be separated into concordant and personal patterns. We suggest that the concordant‐pattern cytokines may be driving the common aspects of the response to this injury type, while the personal‐pattern cytokines reflect the patients’ heterogeneous circumstances. TNFα and IL‐10 showed simultaneous kinetics but opposed changes, supporting the hypothesis that the inflammatory and anti‐inflammatory arms of the immune response are activated together. Finally, the IL‐1RA, GCSF, MIP‐1β and MCP‐1 changes over time show a networked correlation structure, suggesting they may play an important role in the immune response to this injury type.

## CONFLICT OF INTEREST

The authors declare that no conflict of interest exists related to this work.

## AUTHOR CONTRIBUTION

UT and FD contributed equally to this work. All authors participated in the design of the work and the preparation and review of the manuscript. FD collected most of the biological samples and clinical data, with some help from UT. UT performed the laboratory experiments, primary data analysis and first draft of the manuscript. UT, FD, PVvH and SS performed the data analysis and interpretation.

## Supporting information

Fig S1‐S4

## Data Availability

The data sets generated and analysed during the current study are available from the corresponding author upon reasonable request.
