# Supplementary material for: Longitudinal patterns of cytokine expression at the individual level in humans after laparoscopic sleeve gastrectomy
Source: J Cell Mol Med. 2020 Apr 26;24(12):6622–33. doi: 10.1111/jcmm.15309 (PMC7299711; doi:10.1111/jcmm.15309)
Supplement: Supplementary file 1 — Fig S1‐S4 [file JCMM-24-6622-s001.docx]

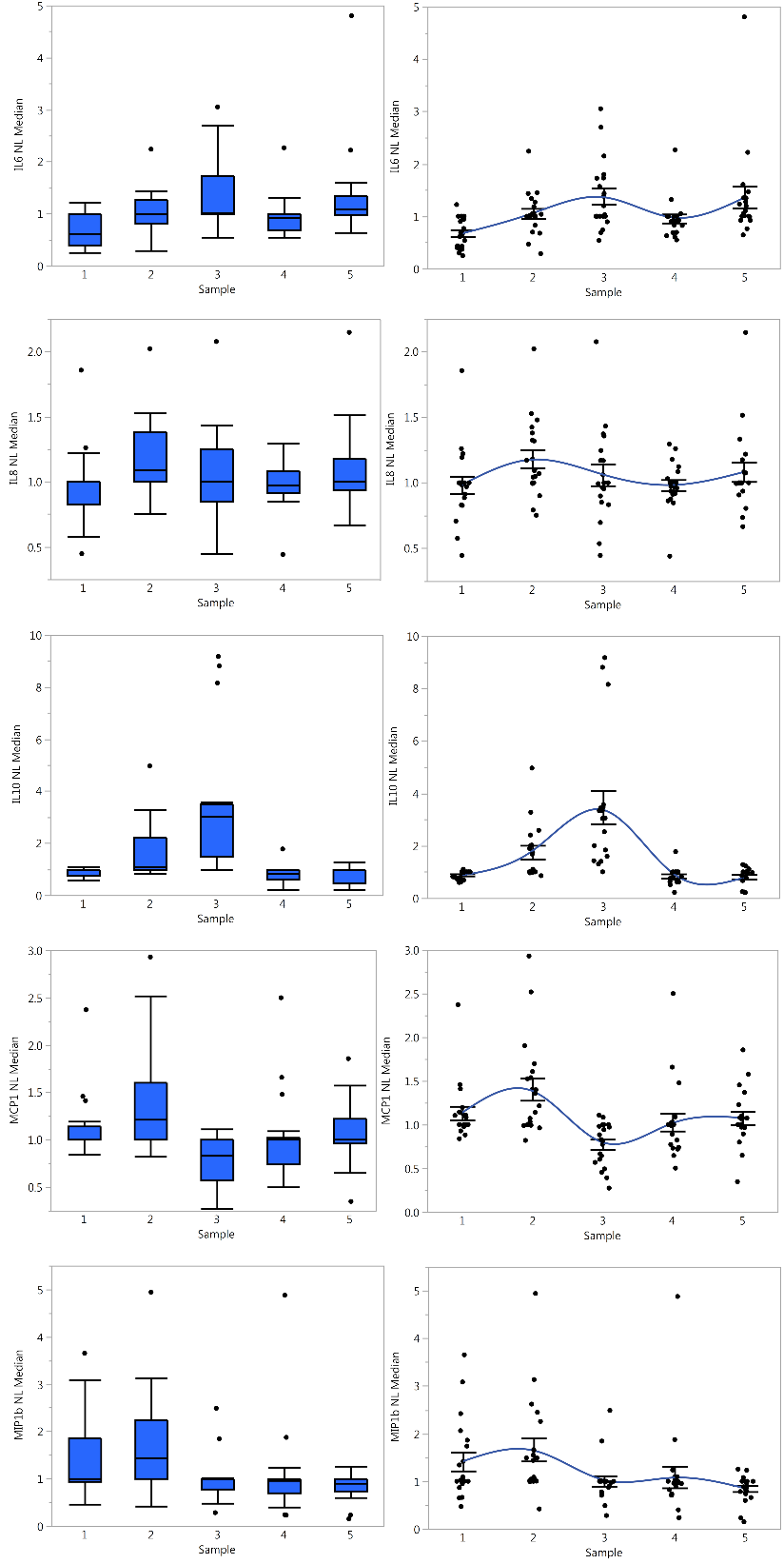

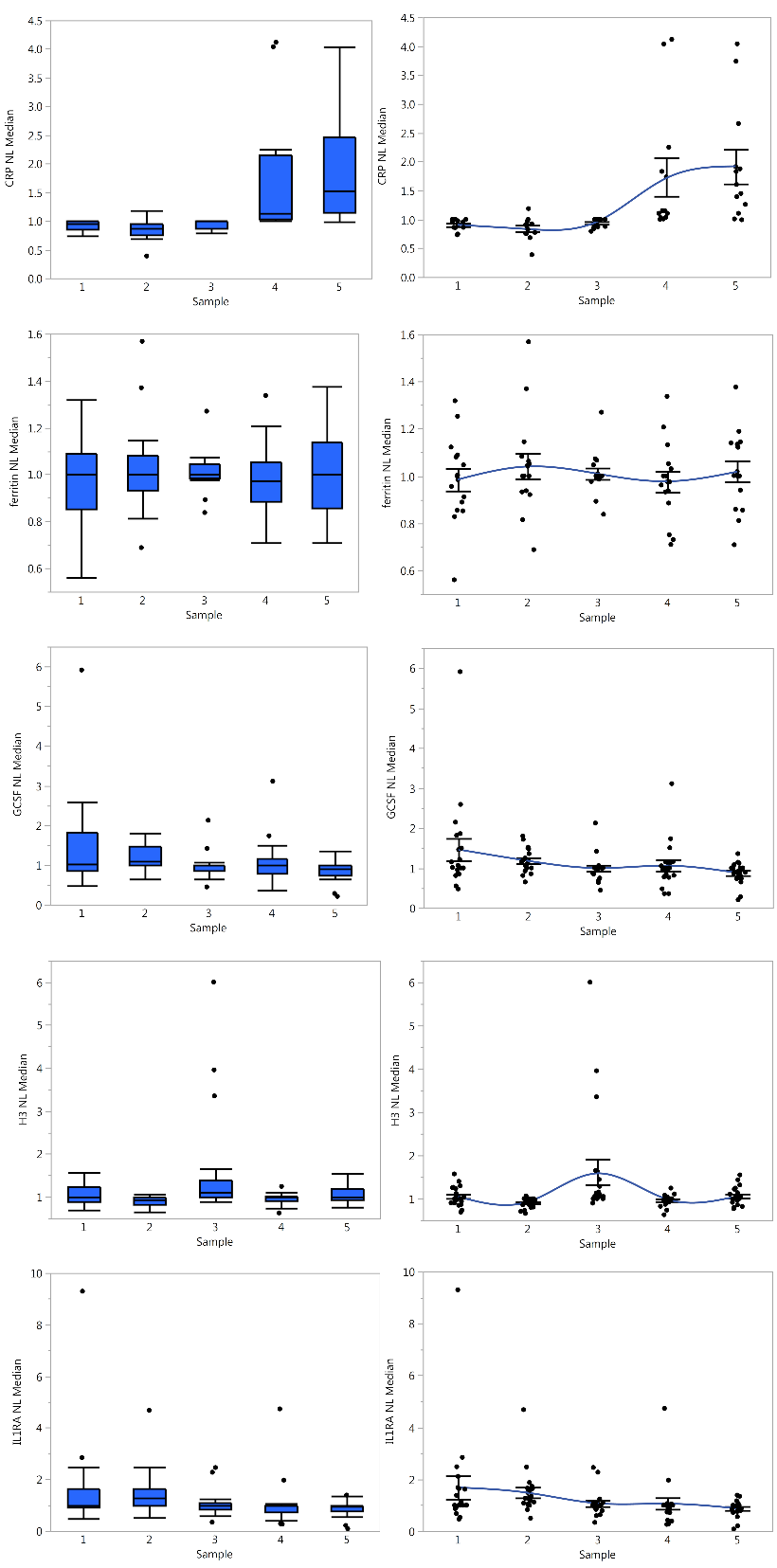


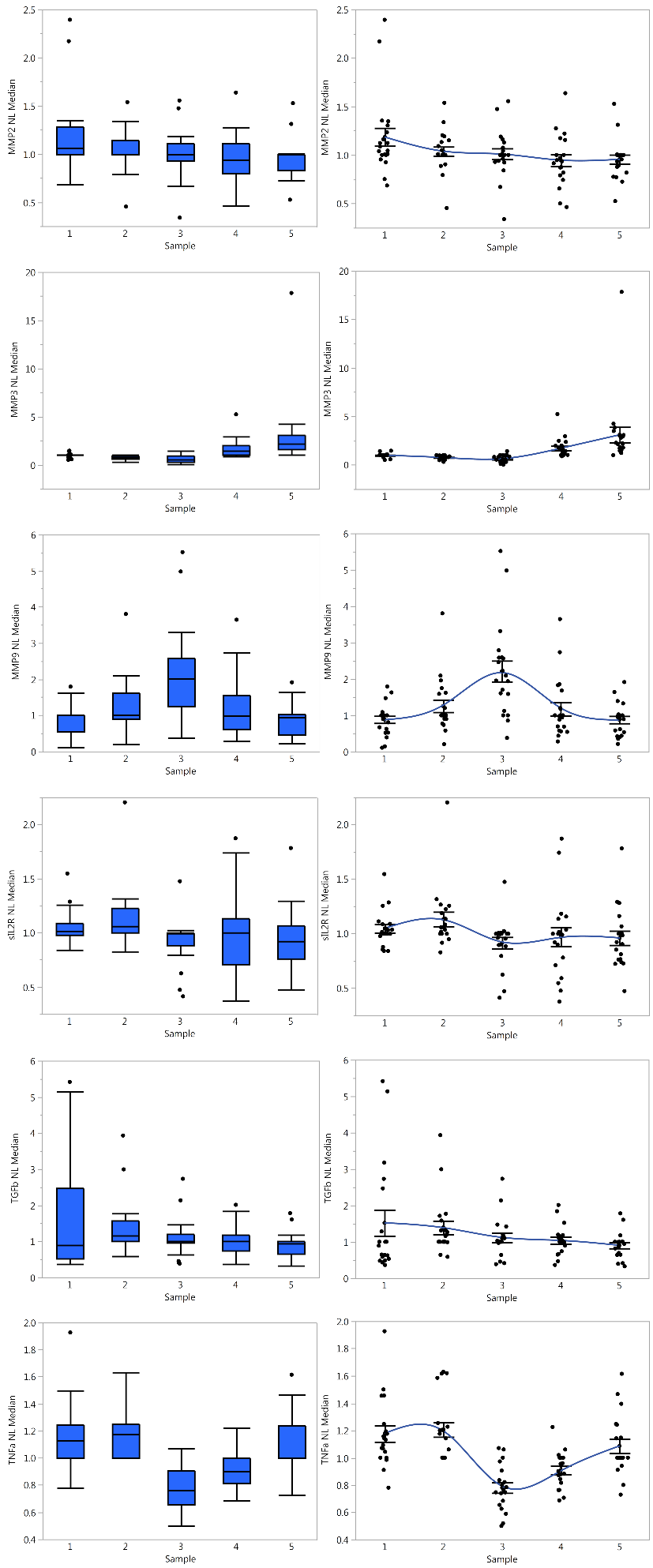

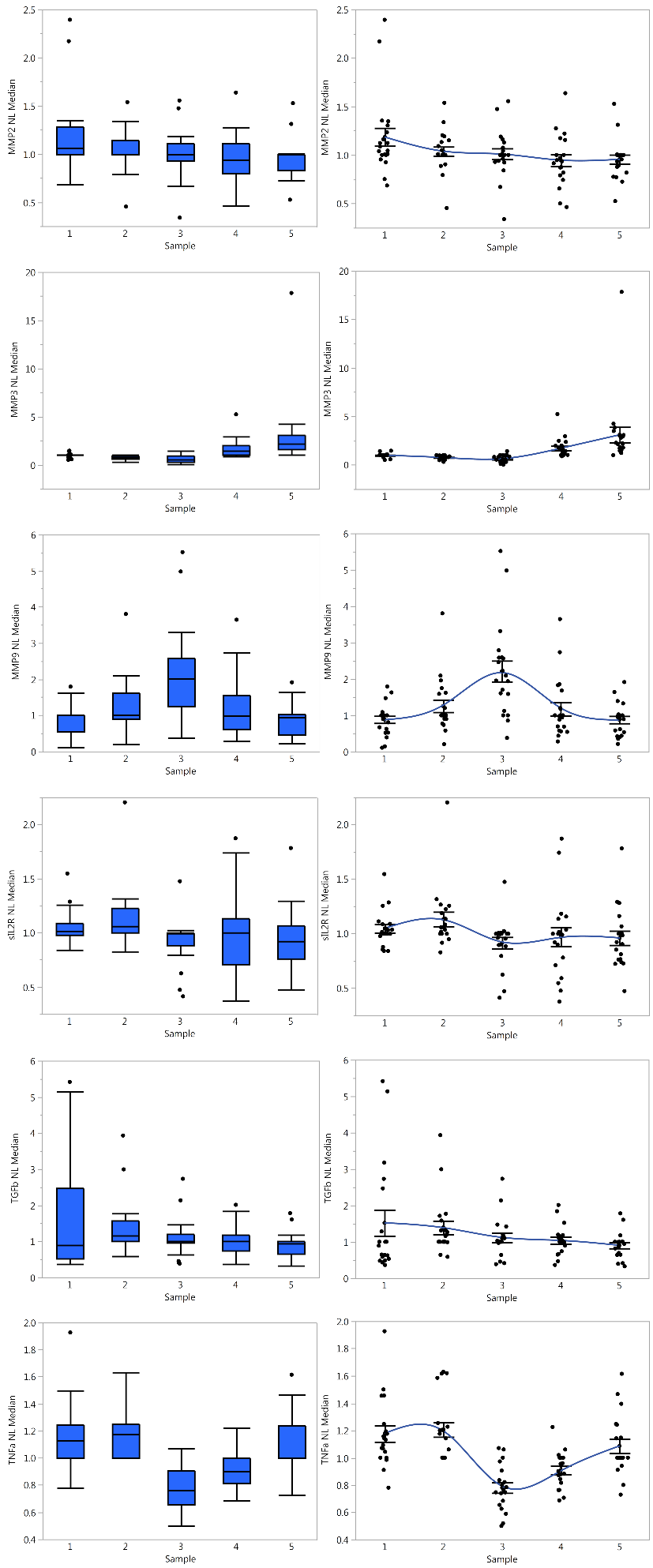


**Supplemental figure 1 - distribution of all cytokines over time**

This supplemental figure is formatted as in figure 1, but shows the data for all of the cytokines. The distribution of the different cytokines is shown over the five sampling times. In the left column, they are shown as outlier box plots. The box is bound by the 3^rd^ quartile at the top and the 1^st^ at the bottom, encompassing the interquartile range; the line inside the box represents the median; the whiskers are drawn extending 1.5 times the interquartile range from the top and bottom of the box; outliers beyond this range are shown as dots. In the right column all of the measurements are shown as dots, with a fitted curve passing through the averages, connecting them; the whiskers encompass the 95% confidence interval of the mean. The values of the cytokines have been row normalized using the median of each patient’s five samples, in order to compare between patients and across different experiment batches.

**Supplemental figure 2 – full results of the mixed models for cytokines, vital signs and clinical laboratory results**


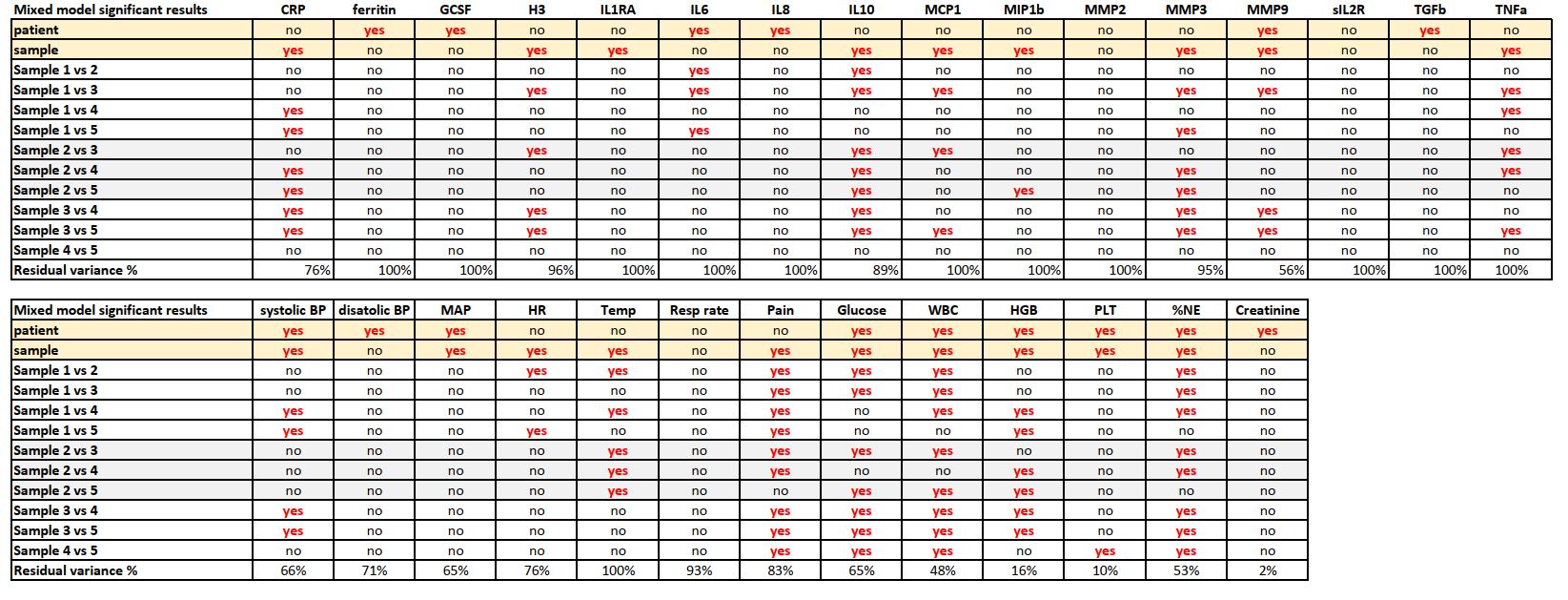
 (see next page) All of the parameters appear as columns. Statistically significant results (p<0.05) appear in red as “yes”. The “patient” and “sample” rows indicate whether these effects significantly contributed to the mixed model; for detailed explanations, see table 1. The rows with paired sample names indicate whether the model found statistically significant differences between those two sampling times for the given parameter. The “residual variance %” row shows how much variance the model cannot account for; for detailed explanations, see table 1.


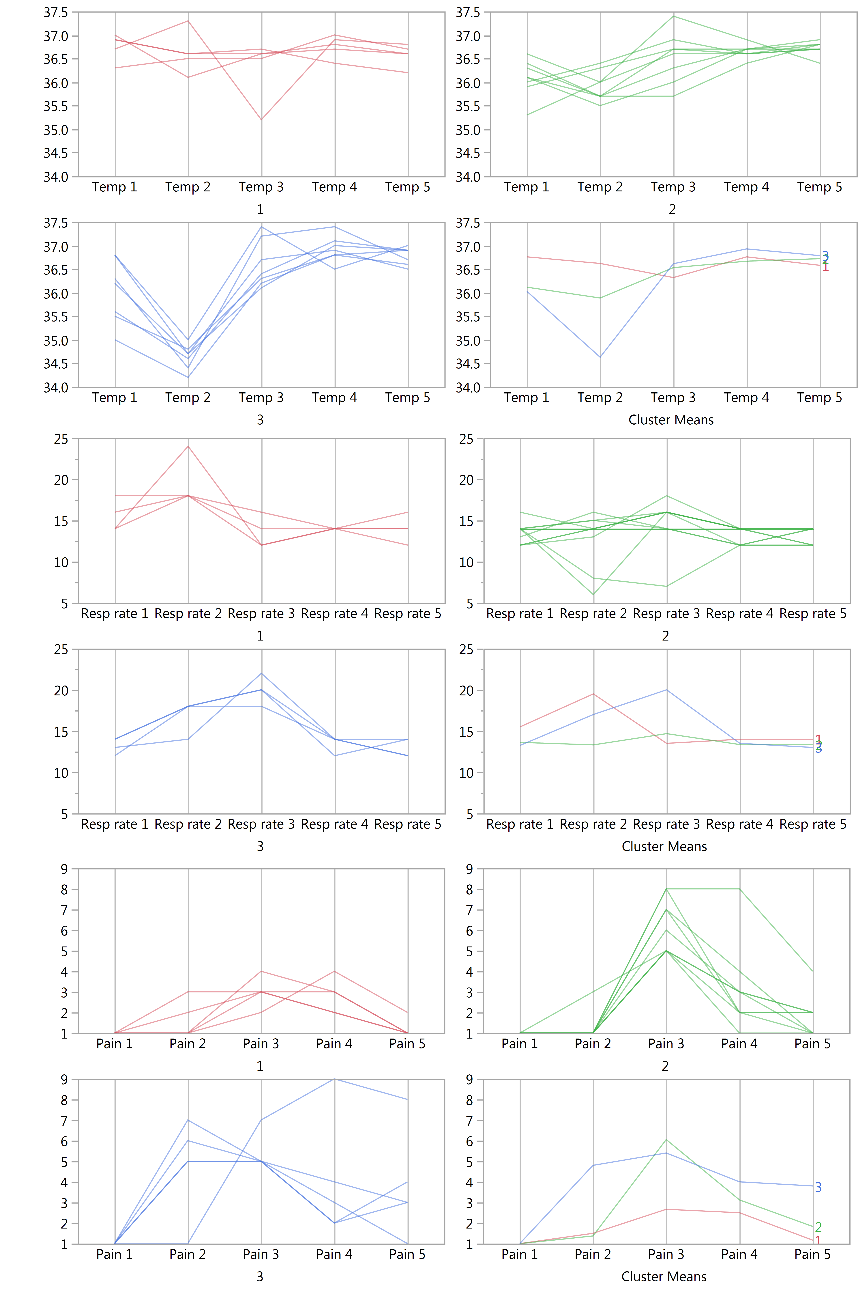

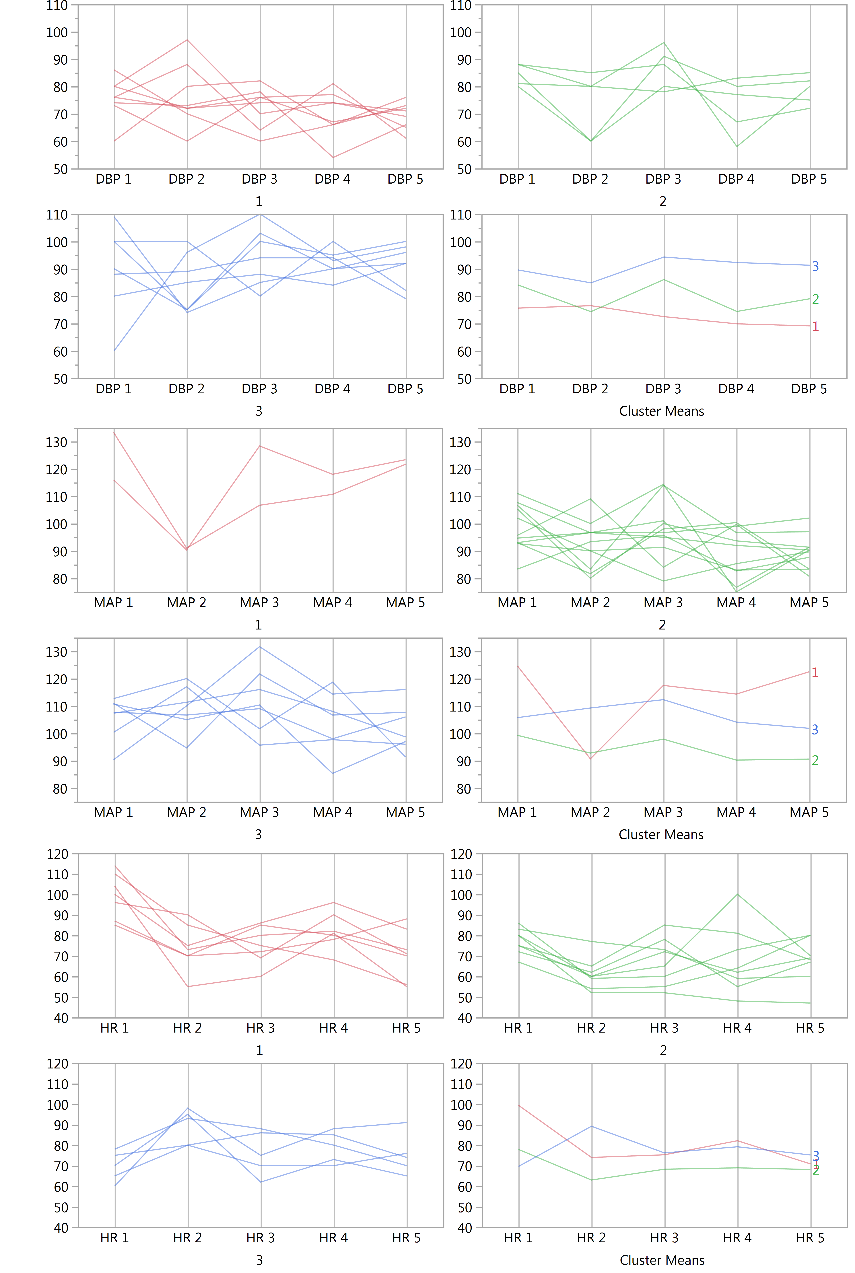


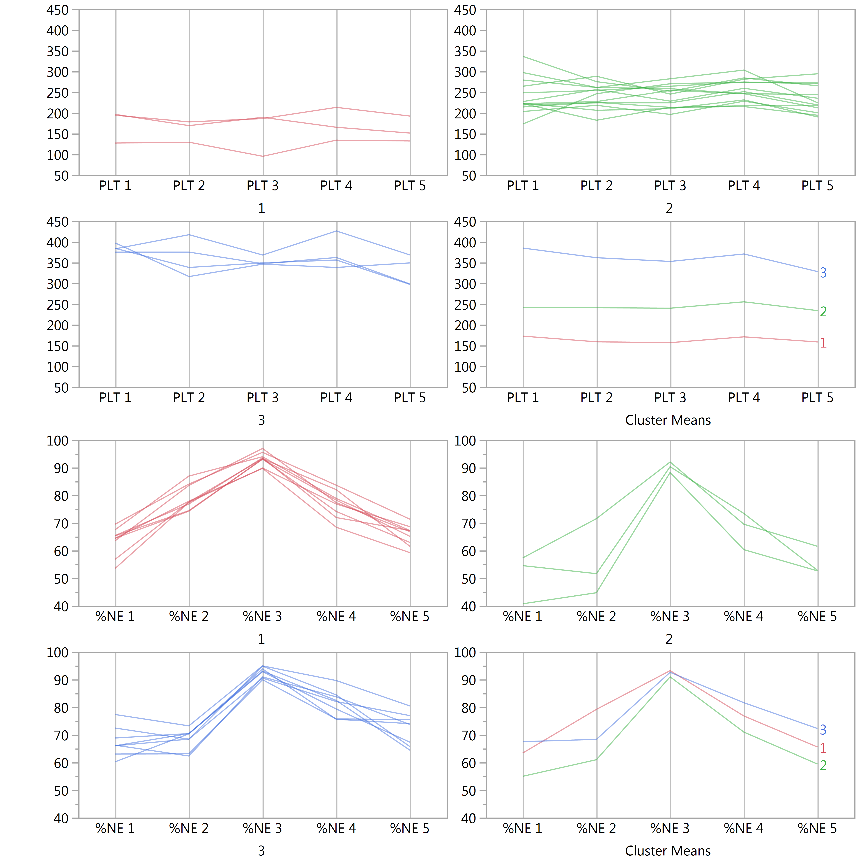

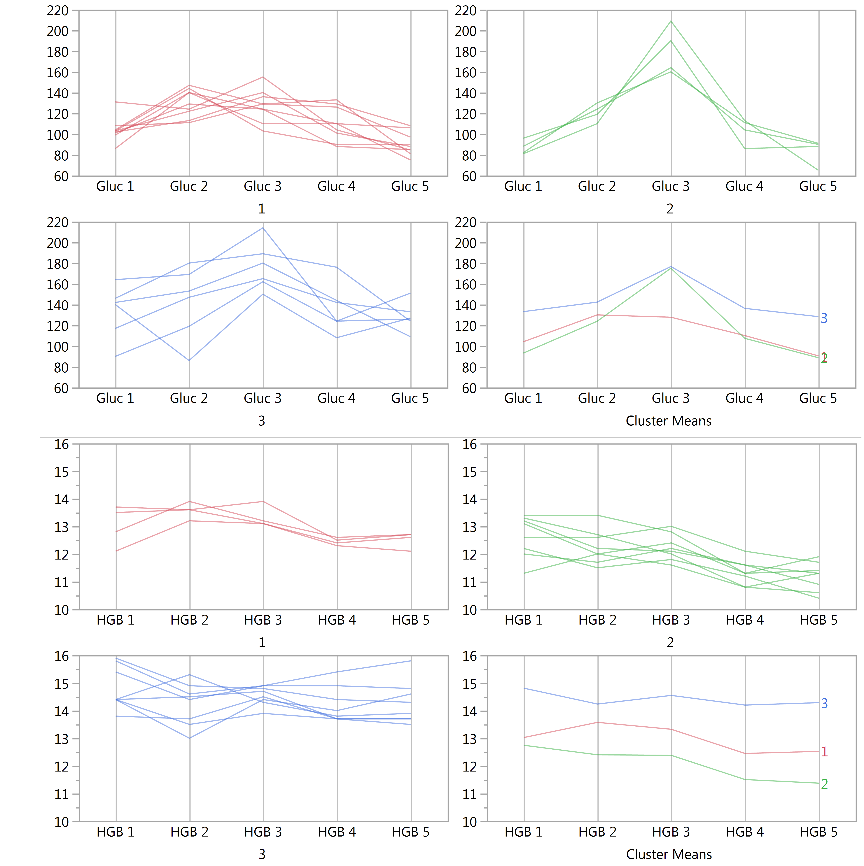


**Supplemental figure 3 – vital signs and clinical laboratory clustering**

This supplemental figure is formatted as in figure 3, but shows the data for all of the vital signs and clinical laboratory results. The parameters were plotted so that the “Y” axes represent the absolute values measured and the “X” axes represent the five sampling times. Every line represents a single patient. The red, green and blue plots represent the three different clusters found, numbered 1 to 3, for every parameter. Each parameter was clustered separately, therefore the patient allocation in the clusters is not the same for the different parameters (i.e. the patients in cluster 1 for the SBP are not necessarily the same as in cluster 1 of the WBC). The cluster means represent the averaged response of each cluster. The parameter’s units are: BP in mmHg, HR in beats per minute, temperature in degrees Celsius, respiratory rate in breaths per minute, pain in a scale from 1 to 10, glucose in mg/dL, WBC in 10^9^/L, HGB in g/dL, PLT in 10^9^/L.


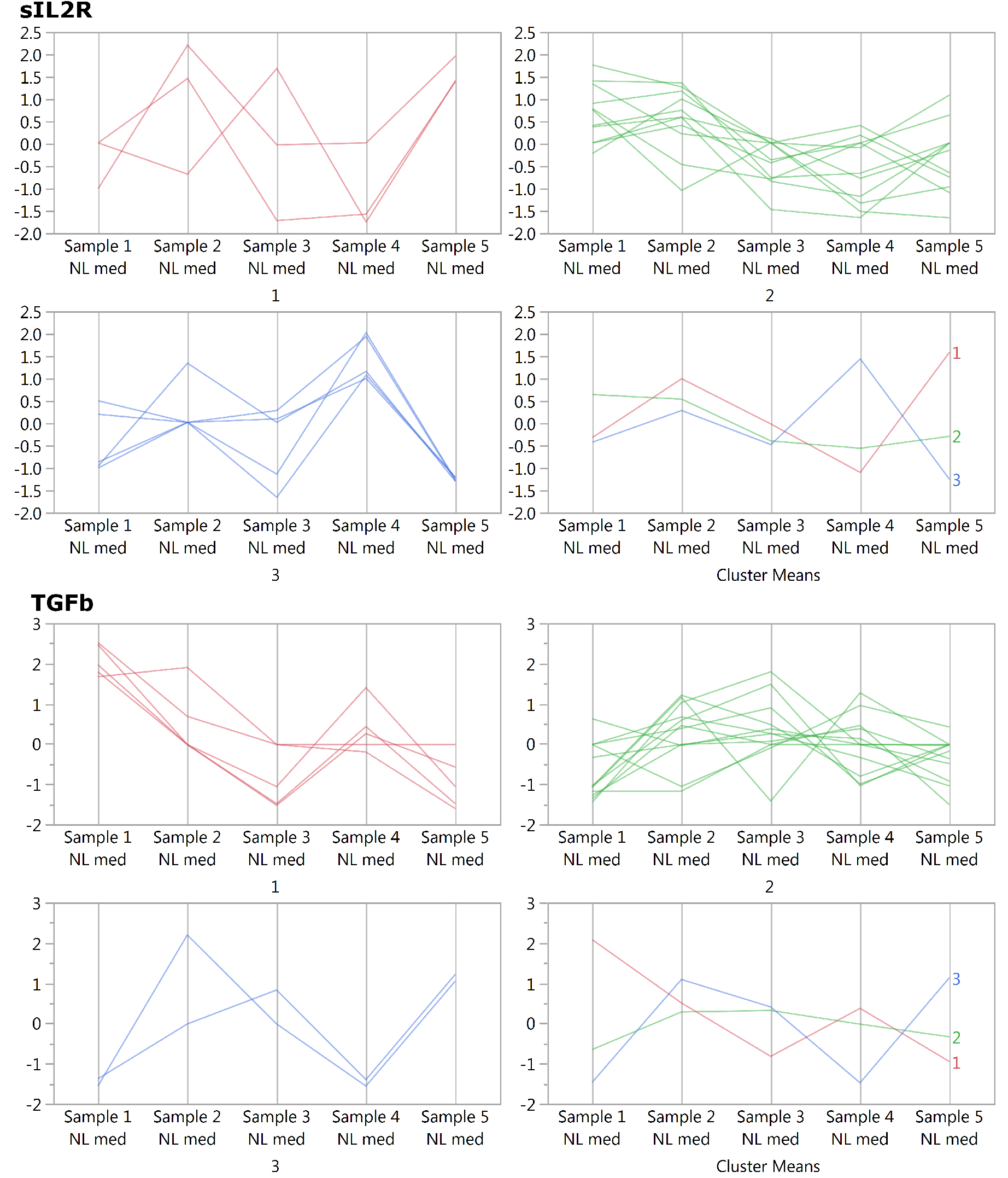

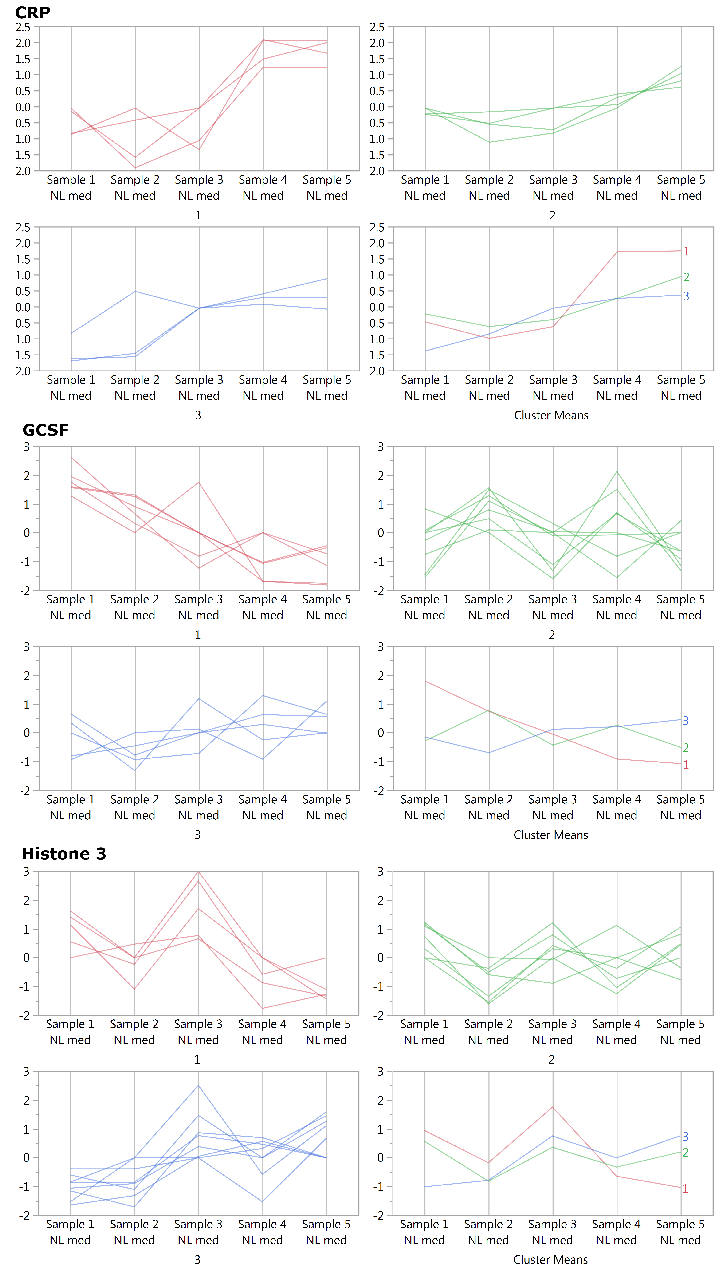


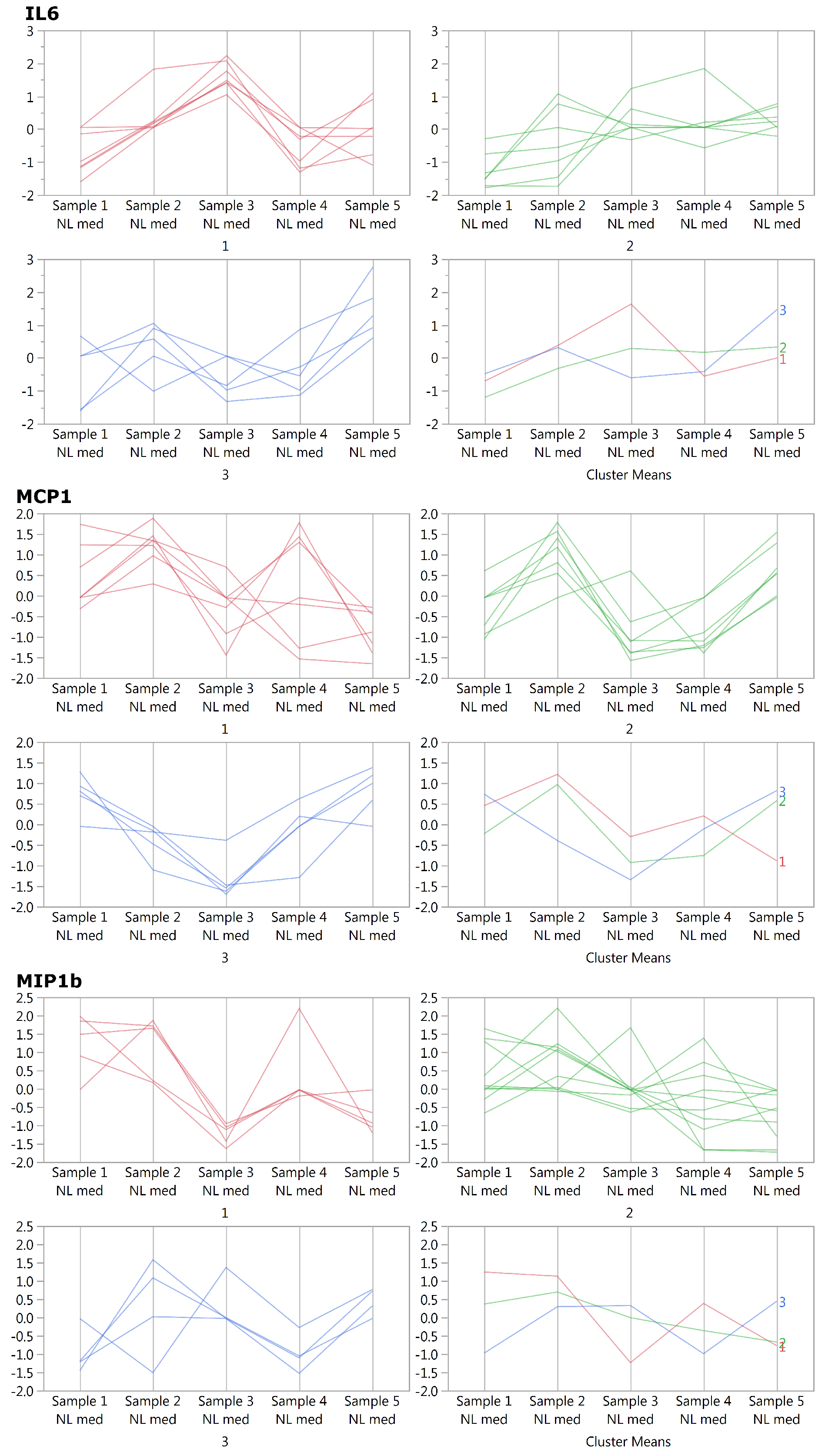


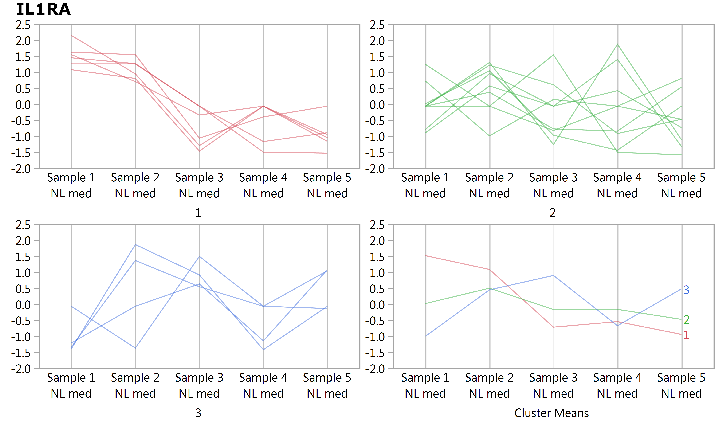


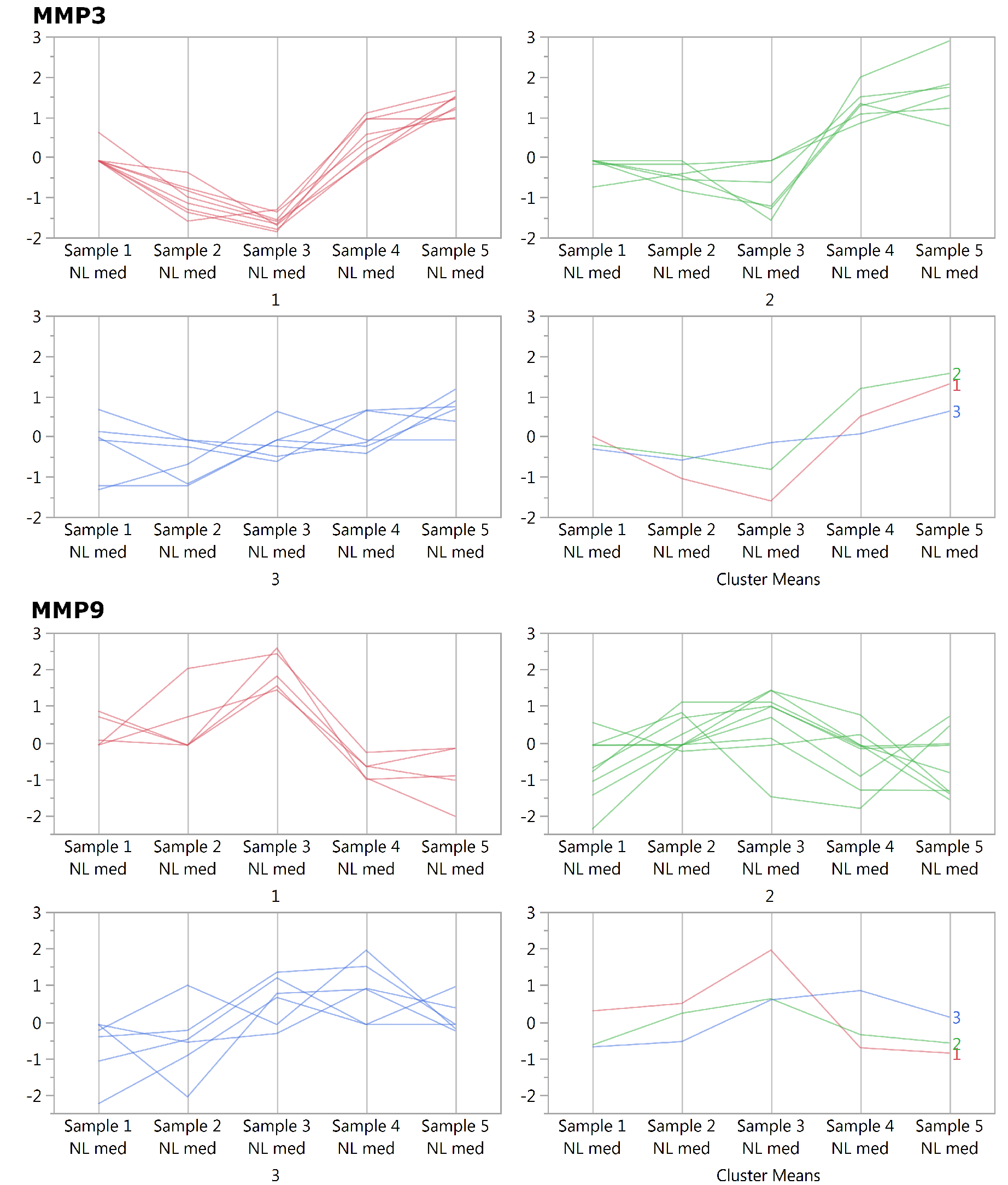


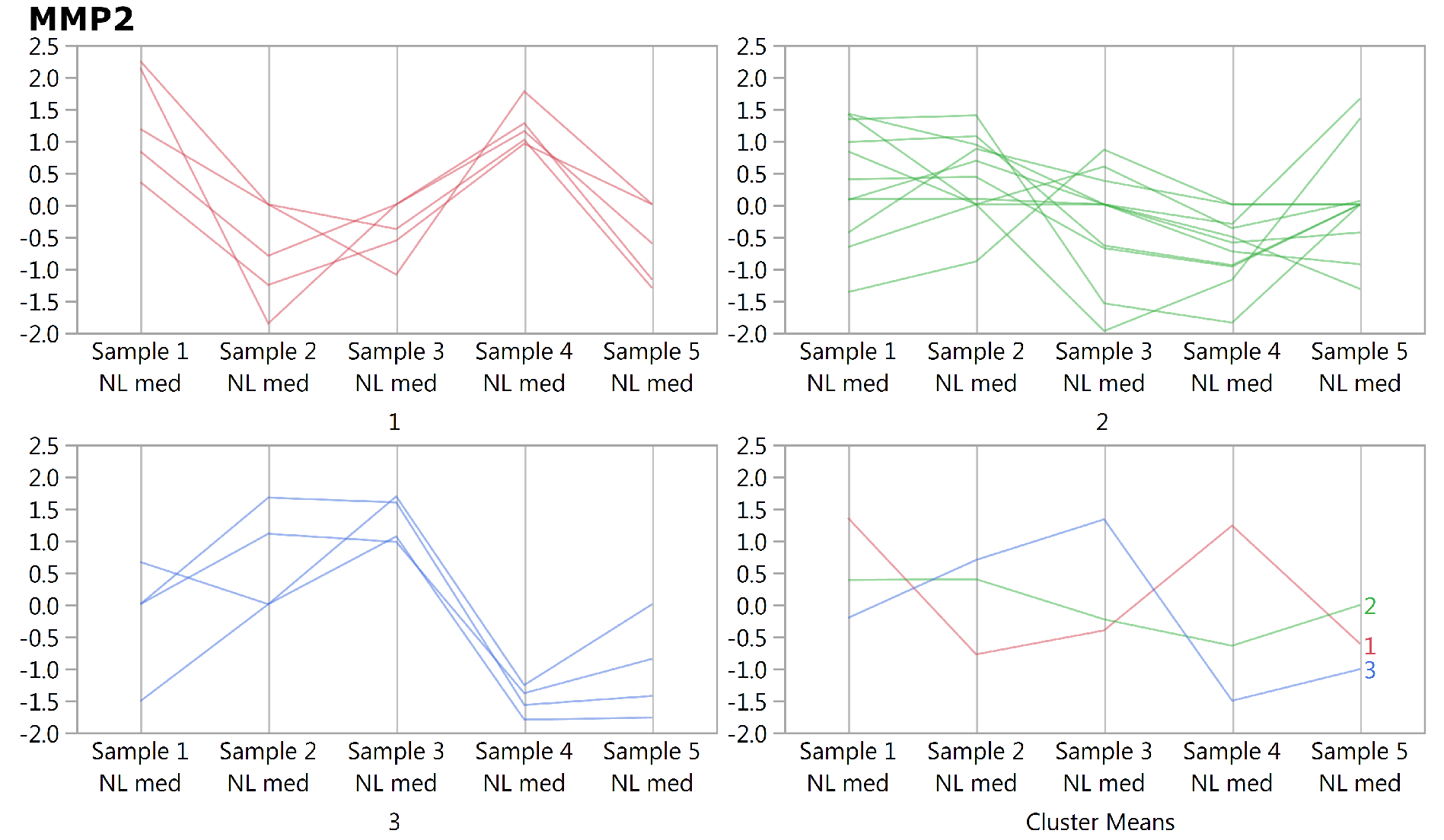


**Supplemental figure 4 – Cytokine clustering**

This supplemental figure is formatted as in figure 4, but shows the data for all of the cytokines. The parameters were plotted so that the “Y” axes represent the median-normalized cytokine values, per patient, and the “X” axes represent the five sampling times. Every line represents a single patient. The red, green and blue plots represent the three different clusters found, numbered 1 to 3. Each cytokine was clustered separately, therefore the patient allocation in the clusters is not the same for the two cytokines (i.e. the patients in cluster 1 for IL6 are not necessarily the same as in cluster 1 of MMP3). The cluster means represent the averaged response of each cluster. Normalization per-patient allows comparing the fold-change in expression over time between the different patients but does not show absolute levels.
